# The equity implications of an expanded health and wellbeing role for housing associations

**DOI:** 10.1016/j.puhip.2023.100355

**Published:** 2023-01-14

**Authors:** A. Hjelmskog, I. Deas

**Affiliations:** a MRC/CSO Social and Public Health Sciences Unit, University of Glasgow, Berkeley Square, 99 Berkeley Street, Glasgow, G3 7HR, UK; b University of Manchester, MSc, University of Strathclyde, The University of Manchester, Oxford Road, Manchester, M13 9PL, UK

**Keywords:** Social housing, Health inequalities, Devolution, City-regional governance, Austerity

## Abstract

**Objectives:**

in light of the acknowledged relationship between housing circumstances and health outcomes, the research explored the implications of the diversifying role of housing associations, considering the extent and form of engagement with the health sector and the potential repercussions for inequalities.

**Study design:**

the research was based on a single case study of the Manchester city-region, chosen to provide a way of considering the role of recently agreed devolved governance and funding arrangements in respect of housing and health.

**Methods:**

primary qualitative data were assembled via a programme of semi-structured interviews with housing and health policy actors and direct observation of six quarterly meetings of a housing-health steering group established as part of new devolved governance arrangements.

**Results:**

the findings reveal a perception among housing managers that the reorientation of housing association services to offset the rationalisation of mainstream provision risks exacerbating inequalities. Interview and observational data suggest that the diversification of housing association activity may have begun to erode the sector's ability and willingness to provide affordable housing on a universal basis to those in need.

**Conclusions:**

The growing non-landlord functions of some housing associations can act as a deterrent to the allocation of housing to applicants with complex (and expensive) needs. This reinforces the increased selectivity in housing association stock allocations, linked to marketization and the increasingly commercial outlook of some providers. Further inequalities may be engendered because while tenants can benefit from the extended housing associations services, others continue to depend on a weakened statutory sector.

## 1 Introduction

The importance of the relationship between housing circumstances and health outcomes is well documented [[1], [2], [3]]. Recent years have witnessed the continuing growth of the housing association sector, extending a long-term process in which publicly-owned council housing has started to be replaced as the principal form of social housing in the UK. Housing associations in England ‘are independent societies, bodies of trustees or companies established for the purpose of providing low-cost social housing for people in housing need on a non-profit-making basis [, where any] … trading surplus is used to maintain existing homes and to help finance new ones’ [4]. As such, they have both social and commercial functions to fulfil.

Previous research has identified the increased tendency of the expanded housing association sector to offer additional services beyond the core landlord function [[5], [6], [7], [8]], necessitating more partnership working and better integration between the housing and health sectors [9]. But while there is a longstanding, transdisciplinary seam of research exploring how housing functions as a social determinant of health [10], understanding of the implications for health inequalities posed by the expansion of the housing association sector, and its expanding remit, remains in its infancy.

This paper reports on research aimed at exploring the ramifications of the changing role of housing associations for public health. Much of the previous discourse on housing-health interconnections has focused primarily on linear, individual- or household-level relationships between housing circumstances and health [11] ^1^ [12]. The research detailed in this paper employed a different approach, assessing the housing-health relationship in relation to the wider population health gradient and employing qualitative methods to examine the potential implications for health inequalities posed by the changing role of housing associations.

## 2 Methods

The findings outlined in the paper are drawn from data collected as part of a case study of the evolution of housing associations' health-focused activities in Greater Manchester, the first UK city-region to embark upon a systematic programme of devolution of health and social care [13]. Data were collected in 2018-19 via a programme of 37 semi-structured interviews with key stakeholders, including housing professionals, public health and clinical commissioning staff. Further qualitative data were assembled through direct observation of six quarterly meetings of the Greater Manchester Housing Providers’ Health Steering Group. Thematic analysis of the data was undertaken iteratively, following the six-step process recommended by Braun and Clarke: 1) familiarisation; 2) coding; 3) generating themes; 4) reviewing themes; 5) defining and naming themes; 6) writing up [14].

The process of institutional and policy reform linked to the Greater Manchester city-region has attracted sustained research interest [[15], [16], [17], [18], [19], [20]]. In this paper, we add to the existing literature on the governance of Manchester by exploring devolved institutional and policy arrangements for the reoriented and expanded housing association sector, its interactions with health and social care providers, and the broader implications for health inequalities.

## 3 Results

The construction of new city-regional institutional structures for Greater Manchester has attracted sustained attention linked to what some commentators view as the city-region's status as a pioneer in sub-national governance and policy innovation [21,22]. These reforms have also prompted a sustained critique, highlighting the limited degree to which rising socio-spatial inequalities have been addressed, the ineffectiveness of mechanisms for public accountability, and the emergence of intra-metropolitan political tensions connected to what critics argue is a preoccupation with economic growth in the conurbation core [[23], [24], [25], [26], [27]].

Greater Manchester's housing providers have been formally incorporated in the city-region's devolved governance structures through a Memorandum of Understanding [28]. Since devolution of the £6 billion annual Health and Social Care budget in 2016, the city-region has embarked on a period of public service reform and health and social care devolution, intended to prioritise ‘wellbeing, prevention and early intervention’ [29]. Greater Manchester is pursuing a health-in-all-policies approach [30], yet the ongoing austerity context and anticipated budget shortfalls for public services create challenges for health improvement goals, limiting the transformative potential of governance reforms [12,31,32].

### 3.1 The expanding remit of the social housing sector: the challenges of additionality and austerity

The housing providers canvassed for this research reported increasingly fulfilling additional, ‘non-landlord’ roles within their communities. This involved providing health and social care services for the first time, sometimes in partnership with other agencies but in other instances comprising services delivered by the housing organisations themselves. Data from the programme of interviews revealed two principal drivers for the diversifying role of housing associations and their involvement in health and social care services. Firstly, housing providers justified the broadening of their activities with reference to wider health and social care policy concerns, such as the increasing emphasis on community-based provision. Housing staff argued that ‘if we're looking at changing the focus of health from hospitals, from institutions, into community settings, [then] we're in a good position to do that’ (Interview, GM3).^1^ Second, housing associations felt compelled to focus more resources on non-housing services in order to offset central government's programme of austerity and the dwindling resources available to other public sector organisations, responding to the increased demand for social care, the escalating cost of meeting the health needs of an ageing population, and the specific needs of a tenant base containing large numbers of impoverished households.

The result of these factors is that the activities of housing associations are increasingly varied, and include interventions targeted at the medical or clinical needs of their tenants, as well as initiatives designed to target the wider social determinants of health (see Table 1).

**Table 1:** Non-housing services offered by Greater Manchester housing associations.

Type of service	Description/Rationale	Selected GM examples (Housing provider in brackets)
Sheltered and Extra Care Schemes	Accommodation for tenants with additional support needs (for example, due to age, disability, mental health), providing homes and varying degrees of care	Limelight Old Trafford (Trafford Housing Trust/L&Q) Morris Court Manchester (Irwell Valley Homes)
Aids, Adaptations, Technology Enabled Care (TEC)	Services that offer adjustments to tenants' homes that will make them easier or safer to live in e.g. for those with reduced mobility, or on-call support	Astraline (Johnnie Johnson Housing)
Social Care	Direct provision of care from the housing provider, intended to join up the accommodation and support requirements of tenants, and to generate additional income for the housing association	Trustcare (Trafford Housing Trust/L&Q)
Food security e.g. food banks, food pantries, holiday kitchens	Rising food insecurity, resulting from higher food prices (along with other essential costs) and lower incomes for many households has led to many different initiatives from housing associations to improve the quality and quantity of food available to their tenants	Real Food Wythenshawe (Wythenshawe Community Housing Group) Your Local Pantry (Stockport Homes) Quids In Food Club (Southway Housing Trust)
Mental health support e.g. counselling, occupational therapy, peer support groups	Mental health problems, and reduction of early intervention or low-level support services at the Local Authority level, are reported to be a significant challenge for housing officers, which some organisations are attempting to rectify internally	Talk, Listen, Change (Stockport Homes) Tidy Homes Tidy Minds (Southway Housing Trust)
Debt and Financial Advice and Support	Increased support from many organisations to support tenants with welfare reform, benefit cuts/caps, transitions to Universal Credit etc.	Breathing Space (One Manchester)
Homelessness support	Several housing associations are commissioned to provide or support their local authority's homelessness services, particularly when the local authority's resources are overstretched	Petrus (Regenda Group)
Employment and skills, volunteering, training, apprenticeships	Supporting tenants into (better) paid employment is a priority for several housing organisations to protect against welfare reform and benefit cuts, for both their tenants' wellbeing and the commercial interests of the housing provider	YES Manchester (Northwards Housing) Barrier-busting, Skills for Life and Work Programme, Work Clubs (Bolton at Home) Step Ahead Employment Programme (Rochdale Boroughwide Housing)
Libraries and Community Centres	Public facilities as risk of closure or reduced hours are taken over (fully or partially) by housing associations, and supported by their staff, facilities & volunteers	The Place at Platt Lane (One Manchester) Old Trafford Library at Limelight (Trafford Housing Trust/L&Q)
Hospital discharge, Step-Up Step-Down facilities	Intended to reduce delayed transfers of care (DTOC), or ‘bed-blocking’, by offering housing sector support to hospital clinical discharge or A&E teams	Hospital 2 Home (First Choice Homes Oldham) A&E to Home (First Choice Homes Oldham) Neighbourhood Apartments (Johnnie Johnson Housing)

Housing providers view their expanding role as a necessary response to the need they have identified among their tenants, linked partly to a perception that help should be increased or extended, but more commonly to the loss or reduction of services provided by other organisations. Interviewees drew particular attention to cuts by local authorities, reflecting the extent to which they have been subject to disproportionate reductions in central government funding in comparison to other parts of the public sector [33]. Housing officers ‘are dealing with some incredibly stressful cases, and we're seeing a withdrawal of some services and just struggling to see, where do I refer this person to?’ (Interview, TD1).

While housing staff recognised that the areas they work in ‘have had to make an awful lot of cuts’ (Interview, GM7), there was concern about their ability to provide the resources needed to compensate. Housing associations are independent organisations, separate from the statutory services, but many interviewees felt that ‘we are being left to plug more and more gaps’ (Interview, GM7) and that ‘we are, absolutely, we are the safety net’ (Interview, GM4), ‘the last man standing’ (Interview, TD1). Interviewees observed that ‘when other systems are under pressure, we get swamped’ (Interview, TD9), acknowledging that their expanded role represents substitution, rather than additionality:

‘We’re just trying to fill a gap. What you’ll find with housing associations is we’re filling a gap, that actually the council used to do years ago, and it’s just because they’ve had cuts, it’s not because they don’t care or don’t want to do it, they just can’t do it’ (Interview, GM10).

The increased degree to which housing associations feel compelled to act as a safety net against austerity prompted apprehension among several interviewees. There was often a sense of unease about the individual and institutional capacity to finance, manage and deliver an extended range of services. As one interviewee put it: ‘I couldn't even begin to tell you how much stuff we do’ (Interview, GM9). Housing organisations, it was argued, lacked expertise and familiarity in delivering non-housing services, reinforcing their reliance, at least in the short-term, on external organisations unused to forging partnerships and themselves subject to intensifying budget constraints and shrinking workforces. These additional services were perceived by interviewees as varied and inconsistent. Where housing associations replaced existing provision by other organisations, quality was viewed as a cause of concern. In cases where capacity has been removed from mainstream services,

‘housing providers, to be fair, have continued in a lot of cases to deliver that support. Not necessarily to the same level, but certainly to the best of their abilities, and they should be commended for that’ (Interview, GM11).

The result of cuts to mainstream services is that housing association staff are confronted with a host of tenant needs for which they may not be adequately skilled or resourced. An example cited was mental health services, where housing officers ‘are spending longer trying to deal with those things, without the skills to do it very effectively’ (Interview, GM7). At the same time, demand is increasing, leaving housing associations increasingly faced with the challenge of supporting tenants who have ‘quite complex needs, where it's hard to get a solution for them because they're not eligible for a service, and really, they're not able to manage their tenancy’ (Interview, OD4).

Interview evidence suggests that the substitutive role in service provision played by housing associations had led to perceptions of diminished effectiveness and efficiency, reflecting a lack of institutional and individual skills and experience. This has necessitated the recruitment of more frontline staff, which itself has ‘been quite a challenge’ for some housing associations, because ‘the people that we're employing now have to have so many different skills … Sometimes I think I'm asking too much because the role's really changed’ (Interview, GM2).

While in aggregate terms the housing association sector has assumed multifunctional service responsibilities, some social landlords have resisted an expanded and diversified role. This reflects some of the practical concerns highlighted around recruitment and skills, but also continuing financial pressures because of the higher cost of delivering non-housing services and the quasi-commercialisation of some aspects of core housing provision. Some interviewees noted the increasingly entrepreneurial mindset of some housing association managers, highlighting the dissonance between the commercial imperative of ensuring the financial viability of housing provision, and the professional and personal expectation – reinforced by external exhortation – that housing associations should fulfil a broader service delivery role in line with their historic sense of social responsibility:

‘We’re doing it as a business, there’s definitely a business argument about more sustainable tenancies, people then pay their rent, but you know, we’ve all got a social conscience; we’re all in social housing for a reason’ (Interview, GM10).

The increased risk and responsibility that accompanies service diversification was perceived as a deterrent for some of the housing providers interviewed, particularly for resource-intensive or high-risk endeavours such as the direct provision of social care. One chief executive reported that eschewing diversification into Extra Care (self-contained supported living) had been ‘a strategic decision by [our] board [because] it's very expensive’ (Interview, GM2). Some expressed an unwillingness to assume additional responsibilities, arguing ‘we've got enough on our plate, how and why would we invest [beyond] that?’ (Interview, GM3). Others were more ambivalent about shouldering new responsibilities but were constrained by a lack of resources, either in terms of personnel and a shortage of ‘people working on non-housing issues’ or because they were ‘less well-off than a lot of the others’ (Interview, GM7). Despite these sectoral resource constraints, however, some housing association boards ‘just took the view that, if we're going to make a loss, [building supported housing] is the right thing to do, it's the right product in the right place at the right time, we're going to do it anyway’ (Interview, GM8).

### 3.2 Access, equity and selectivity in the allocation of housing association services

The increasing importance of housing associations as providers of health and social care services has potentially important implications for inequality. Non-housing services provided by housing associations vary quantitatively and qualitatively, depending on the extent to which housing providers choose to diversify their activities and their varying capacity to do so effectively. Further inequality is engendered because many housing associations have opted to restrict their services to existing tenants. This means that in the local areas in which housing associations operate, non-tenants remain dependent on existing agencies, some of which may have reduced or ended service provision. As one interviewee argued:

‘In some senses it’s creating a two-tier system of access for people. If you’ve got a social landlord, you can access [services] through these routes, but if you haven’t … is that fair? Is that equitable?’ (Interview, TD2)

Some providers argue that offering social and health services allows their tenants to satisfy the rising thresholds to qualify for mainstream provision, and/or to shorten or circumvent the long waiting times that would otherwise be involved. The result, however, is to engender inequality in access to services, privileging housing association tenants. For example, one organisation has ‘provided counselling … we've got a contract with [Relate] to provide free counselling because we found that our customers were waiting three or four months if they went to the GP to get counselling’, but if their tenants approach the housing association for counselling ‘they can get it this week’ (Interview, GM10).

Interviewee testimony also suggests increasing inequalities linked not just to the decision of some housing associations to offer expanded non-housing services, but also because of the changing socio-demographic profile of tenants accessing social housing. Housing associations have responded to changes in the social housing grant funding model and the reduction of government funding by taking on more debt, and by increasing their emphasis on ‘higher risk’ development [34]. This has included focusing on potentially more profitable types of housing stock (‘affordable’ rent, shared ownership, market rent and sales of full value properties on the open market), at the same time as reducing new lettings at social rent levels [35]. Reflecting the increased marketization of social housing and the increasingly business-minded outlook of some housing associations, newer housing developments may be more likely to attract more affluent tenants or owner-occupiers. Many housing associations ‘probably still see ourselves as an alternative to private renting’, but through their expansion into market rate or shared ownership housing they have ‘tried to diversify around who we house … we advertise on places like Right Move which is alongside what the private rented sector would do’ (Interview, TD1). The result is that housing associations in areas of high demand are increasingly accommodating more affluent tenants able to exercise choice, while growing numbers of lower income households are reliant on the private rented sector [36,37]. This was viewed by some interviewees as compounding the inequalities resulting from the ‘two-tier’ housing system, as the largest proportion of homes that do not meet Decent Homes Standard are consistently found in the private rented sector [38].

Some interviewees argued that the additional health and social care services offered by housing associations creates a further incentive for selectivity in housing allocations. Alongside rising rent levels, interview evidence suggests that the socio-demographic profile of the tenant base of some housing associations may be changing because more stringent thresholds are being applied (or existing ones enforced more rigorously) as awareness grows about the resources required by tenants with complex needs. In that sense, the health needs of tenants may incentivise exclusionary decision-making practices in some organisations:

‘We have to be very careful who we take on, because we’re very small … if they have mental health issues, we don’t have a huge amount of resources to deal with that, so if they’re not paying their rent, or they don’t have support workers, or don’t understand things, if they have no financial awareness, it’s going to be very difficult, they’re going to be very difficult as tenants. And managing that … would take up way too much of our time’ (Interview, GM19).

Such caution was expressed by several interviewees, but least often among the stock transfer organisations^2^

which typically saw accommodating tenants with complex needs as a key part of their remit, even if their ability to dedicate the appropriate resources was variable. One Greater Manchester authority, which still owns and manages some council housing, noted that the housing associations operating in their jurisdiction have a tendency to ‘cherry-pick’ the ‘best’ prospective tenants, including those with fewer additional or complex needs (Interview, GM5).

Interviewees recognised the risk of cumulative disadvantage for households who face increased barriers to access social housing. ‘People who are in the awful, lower end of private rented sector accommodation … haven't got that safety net of living in a housing provider property’ (Interview, GM13). Moreover, private tenants do not enjoy access to the non-housing services provided by some housing associations,^3^ as one interviewee argued:

‘Once you’re a [social] tenant, you’ve got health initiatives, you can have counselling, we do employment services, you can get a wealth of different services … but if you’re private sector you can’t’ (Interview, GM10).

The limited capacity of housing associations to provide health services risks undermining the sector's core function of providing decent, secure, affordable homes. Unintended consequences for equity may result because of the costs of prioritising health and social support services [39].

## 4 Discussion

Using the example of the housing association sector, and drawing on fieldwork in Greater Manchester, this paper has identified potential consequences and risks for health inequalities arising from the reconfiguration of third sector services to meet gaps in provision left by retrenchment in the welfare state. The evidence presented suggests that the risks of increasing health inequalities are twofold. Not only is the diversification of housing association activity reducing their capacity to act as a universal provider of affordable housing to those in need, but their wider support services are not easily offered universally to the wider population, some of whom are either ineligible or lack ready access to services, leaving them reliant on an increasingly strained system of statutory support.

The housing association sector in Greater Manchester, as elsewhere in the UK, struggles to meet demand [40], exacerbating the differential ability of social groups to secure or retain access to the limited supply of social housing stock. The findings outlined in this paper underline the concern that the poorest applicants to social landlords may be rejected and deemed too risky to house because of their ‘unmet support needs’ [41,42]. The social housing sector lacks the capacity to provide universal (or at least proportionately universal) interventions, instead relying on piecemeal or inconsistent efforts that previous research demonstrates can accentuate inequality [[43], [44], [45]]. The risk for those in housing need, based in areas with no other significant supply of social housing, is failing the affordability (and other) checks many housing associations perform on their prospective tenants [46].

The experience of the housing association sector in respect to growing inequality in access to services echoes that of public service provision more broadly. Interview findings confirm the results of previous research by Hastings et al. [47], which demonstrated that local authority funding cuts are typically felt most acutely in poorer communities via ‘a tightening of eligibility thresholds’, leaving those deemed ineligible to rely on fallback voluntary sector provision. While housing associations with the means and motivation to do so are able in part to offset the removal or reduction of mainstream services, this safety net may not be available for communities in which non-landlord services are less well developed. The increased reliance on housing associations, in the context of larger public expenditure cuts, represents one of the ‘hidden’ effects or ‘legacies’ of austerity, further undermining what remains of universal provision [[48], [49], [50]].

The move from universal to a two-tier service provision exacerbates health inequalities and creates a mutually reinforcing cycle of exclusion when understood in the wider context of austerity. The restricted options in the private rented sector, and the increased reliance on temporary or emergency accommodation, support the view of interviewees that social housing is no longer ‘the tenure of last resort’ (Interview, GM10). There is a risk that a two-tier system of access (to both housing and health and social care services) is contributing to the cumulative (dis)advantage of certain groups and individuals, whose access to support may be determined by luck and location, rather than entitlement or need. This evidence is illustrative of the sometimes strained ‘precarious partnerships’ between state and non-state actors that are a result of actions taken in the UK since ‘government responsibility for addressing inequality was decollectivised’ [51]. To varying degrees, housing associations have taken on some of this responsibility, resulting in a complex and uneven pattern of service delivery that is adding to the challenge of promoting health equity.

While the purpose of the independent housing association sector has historically been ‘to “fill the gap” where the state or market was unable to provide for households in need’ [52], this ‘gap’ is increasingly viewed as including support beyond housing provision. The services provided by housing associations who ‘step in where the state will not’ do not offer an adequate replacement for universal systems. The wider relationship between housing associations and the welfare state is illustrative of continued erosion of the collective principles that once underpinned the strategies of both universal health(care) and a state that provided support ‘from cradle to grave’ [53]. ‘Beveridge systems’, and the accompanying principle that public services should be distributed equitably and according to need, ‘can be seen as systems level approximation of the general universalism paradigm’ [39]. The findings presented in this paper reveal the difficulties that have ensued as the housing association sector struggles to combat accumulated health disadvantage in an equitable way [54].

## Ethical approval

The research was approved by the ethics committee of the host institution (University of Manchester). The participants provided written informed consent for their involvement in the research.

## Funding

Funding was provided by the 10.13039/501100000269Economic and Social Research Council and the North West Doctoral Training Centre (Award no. 1774610), co-funded by Trafford Housing Trust.

Funding is also provided by CSO/MRC (Funder's reference SPHSU20 MC_UU_00022/5).

## Declaration of competing interest

The authors declare that they have no known competing financial interests or personal relationships that could have appeared to influence the work reported in this paper.

## Footnotes

1 Transcription identities, data anonymised. ‘GM’ refers to interviewees from anywhere within the wider city-region, ‘TD’ and ‘OD’ refer to the two detailed case study boroughs of Trafford and Oldham respectively.

2 ‘Stock transfer’ refers to the process of transferring ownership of council housing to a named housing association, usually established to operate within the original borough, retaining its geographic roots and ties to the local community.

3 This view assumes that the provision of non-housing services can be viewed in broadly positive terms, as a form of ‘justifiable paternalism’ that improves the lives of disadvantaged tenants. It is important also to acknowledge more critical perspectives which frame such support as a mechanism for managing tenant behaviour and maintaining the financial viability of social housing providers [39].
