# Supplementary material for: The equity implications of an expanded health and wellbeing role for housing associations
Source: Public Health Pract (Oxf). 2023 Jan 14;5:100355. doi: 10.1016/j.puhip.2023.100355 (PMC10280050; doi:10.1016/j.puhip.2023.100355)
Supplement: Multimedia component 1 [file mmc1.docx]

**Interview Schedule**
